# Phage therapy against *Klebsiella pneumoniae* infections: integrating host–phage immune interactions, therapeutic strategies, and clinical translation

**DOI:** 10.3389/fmicb.2026.1913201

**Published:** 2026-08-07

**Authors:** John K. Giju, Sandhya Padmakumar, Abhijith Pulimoottil Jaykumar, Pradeesh Babu, Aravind Madhavan, Bipin G. Nair, Geetha B. Kumar

**Affiliations:** School of Biotechnology, Amrita Vishwa Vidyapeetham, Kollam, Kerala, India

**Keywords:** antimicrobial resistance, bacteriophage therapy, delivery strategies, host-phage-pathogen immune interactions, *Klebsiella pneumoniae*

## Abstract

The emergence of convergent multidrug-resistant (MDR) and hypervirulent (*hvKp*) lineages has transformed *Klebsiella pneumoniae* into a major global health threat. Frequently associated with hospital-acquired pneumonia, bacteremia, urinary tract infections (UTIs), meningitis, and sepsis, these strains disproportionately affect elderly, immunocompromised, and critically ill patients, resulting in high morbidity and mortality. Their rapid dissemination has further intensified the global antimicrobial resistance (AMR) crisis, highlighting the urgent need for therapeutic strategies beyond conventional antibiotics. Although bacteriophages have demonstrated considerable potential as antibacterial agents, their successful clinical application depends on factors extending beyond bacterial eradication alone. This review critically integrates current evidence on therapeutic efficacy, host–phage immune interactions, delivery optimization, phage–antibiotic synergy, phage-derived therapeutics, AI-assisted phage discovery, and translational considerations within a unified framework for *K. pneumoniae* infections. It further examines how these interconnected determinants collectively influence therapeutic outcomes and discusses emerging strategies for precision phage therapy. By adopting this integrated translational perspective, the review identifies the key factors governing successful clinical implementation and provides a framework for advancing bacteriophage therapy against multidrug-resistant *K. pneumoniae* infections.

## Graphical abstract

**Figure:**
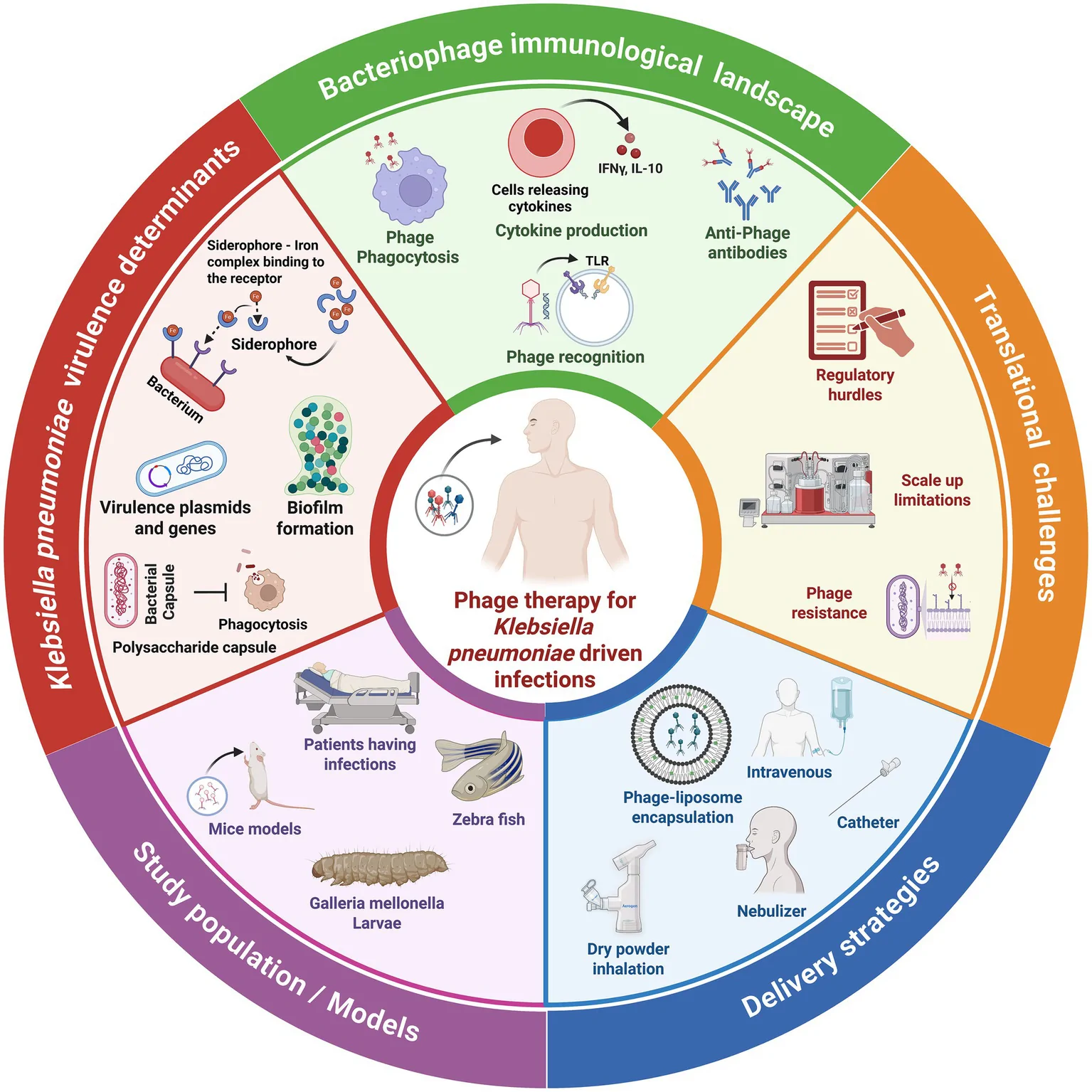
Graphical abstract illustrating the major aspects of bacteriophage therapy against *Klebsiella pneumoniae*-associated infections.

## 1 Introduction

Antimicrobial resistance (AMR) has become a major global health challenge, with ESKAPE pathogens accounting for a substantial proportion of healthcare-associated infections (Pipitò et al., 2025; Gebeyehu et al., 2026). Among them, *Klebsiella pneumoniae* is recognized by the World Health Organization (WHO) as a high-priority pathogen due to the emergence of multidrug-resistant (MDR), extended-spectrum *β*-lactamase (ESBL)- and carbapenemase-producing strains, together with enhanced virulence traits, causing severe infections including pneumonia, urinary tract infections, wound infections, meningitis, and bacteremia. This dangerous convergence of resistance and virulence in clinical isolates underscores the urgent need for alternative therapeutic strategies to effectively combat *K. pneumoniae*-associated infections (Itani et al., 2024; More et al., 2025; Li et al., 2025).

Lytic bacteriophages have re-emerged as promising alternatives for treating multidrug-resistant bacterial infections owing to their targeted antibacterial activity, self-amplifying nature, favorable safety profile, and reduced risk of stable cross-resistance (Kou et al., 2024). Despite their therapeutic potential, the interaction between bacteriophages and the host immune system remains an important consideration for successful phage therapy. Bacteriophages modulate innate immune responses through alterations in phagocytic activity and cytokine production, whereas adaptive immune responses, particularly the generation of phage-neutralizing antibodies, may limit phage persistence and bioavailability *in vivo*. Findings from *in vitro* and murine studies highlight the complexity of these host–phage interactions and underscore the need for a better understanding of their influence on optimizing phage-based therapeutic interventions (Van Belleghem et al., 2018; Berkson et al., 2024).

Despite the growing body of evidence supporting bacteriophage therapy against *Klebsiella pneumoniae*, successful clinical translation extends beyond demonstrating antibacterial efficacy alone (Bris et al., 2025; Pirnay et al., 2024; Pires et al., 2020). Critical determinants such as host–phage immune interactions, therapeutic delivery strategies, phage-derived enzymes, and translational challenges have been investigated extensively in individual studies; however, these factors are often discussed independently (Van Belleghem et al., 2018; Bolsan et al., 2024; Kim et al., 2025; Moon et al., 2025).

Consequently, their collective influence on therapeutic efficacy, clinical applicability, and treatment optimization remains insufficiently integrated. Accordingly, this review adopts a translational perspective by critically integrating current evidence on host–phage immune interactions with recent advances in delivery technologies, phage-derived therapeutics, and emerging clinical considerations, interconnecting factors governing therapeutic success, identifying current knowledge gaps, and discusses future directions for advancing bacteriophage therapy against *Klebsiella pneumoniae* infections.

## 2 The pathogenic landscape of *Klebsiella pneumoniae:* virulence to hypervirulence

*Klebsiella pneumoniae* is a clinically important opportunistic Gram-negative pathogen that causes severe healthcare-associated infections, particularly in immunocompromised patients. Its polysaccharide capsule is a major virulence determinant that promotes bacterial persistence and disease progression (Liu et al., 2023; Asri et al., 2021; Ju et al., 2025; Aldali, 2025). From an epidemiological perspective, *K. pneumoniae* represents a major contributor to global infectious disease mortality, accounting for approximately 800,000 deaths annually, with nearly 80% of these linked to antimicrobial-resistant (AMR) strains. The pathogen is also well known for the wide spectrum of infections that they can cause, including respiratory tract infections, bloodstream infections, and intra-abdominal infections, which together account for almost 90% of *K. pneumoniae*-related deaths and the highest age-standardized mortality rates. Notably, the burden of disease is disproportionately higher in regions such as South Asia and sub-Saharan Africa, with the latter reporting the highest mortality rates attributable to AMR-associated *K. pneumoniae* infections. The global distribution and clinical impact of *K. pneumoniae* are shaped by a complex interplay of factors, including healthcare infrastructure, antimicrobial usage patterns, patient demographics, and regional epidemiological variations (Song et al., 2025).

### 2.1 Hypervirulent *Klebsiella pneumoniae*

Another emerging concern regarding the pathogenesis of *Klebsiella pneumoniae* is the emergence of hypervirulent *K. pneumoniae* (hvKp). In contrast to classical *K. pneumoniae* (cKp), hvKp have shown differences in the dynamics of infection, pathogenesis, genetic determinants, etc. While cKp infections are primarily affecting hospitalized patients or immunocompromised individuals; hvKp is capable of causing pathogenesis in immunocompetent healthy individuals, establishing its significance as an emerging threat (Hetta et al., 2025). Later on, it can establish the infection from the primary site of infection, further disseminating towards different organs such as eyes, lungs, and the central nervous system. It is also associated with severe primary extrahepatic infections, including bacteremia, soft-tissue infections, and pneumonia (Choby et al., 2020).

**Figure 1:**
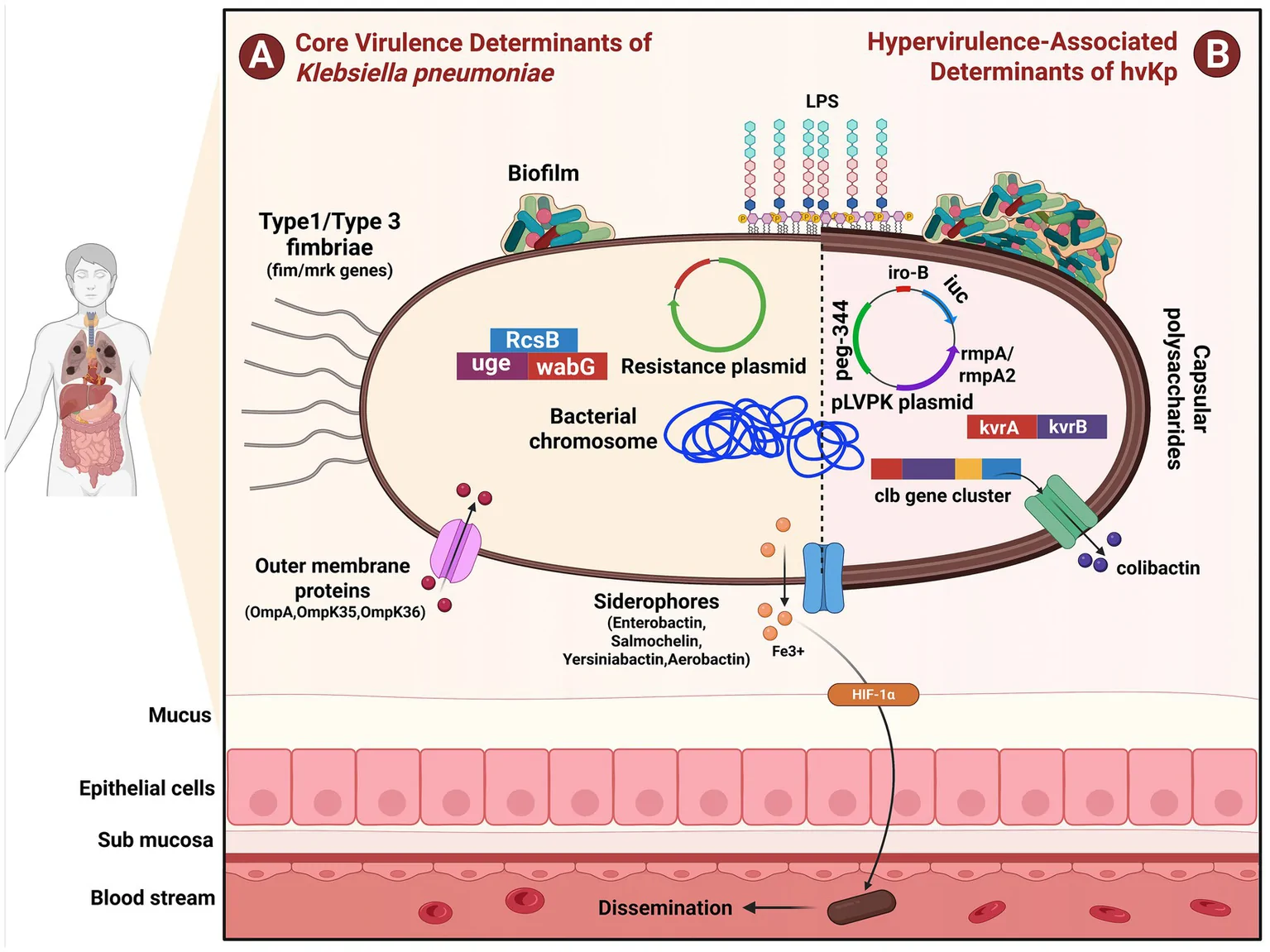
Comparative overview of core and hypervirulence-associated determinants of *Klebsiella pneumoniae* involved in colonization and dissemination.

Large virulence plasmids and chromosomal mobile genetic elements contribute to the hypervirulent phenotype. Among these, the virulence plasmid-associated genes peg-344, rmpA, rmpA2, iroB, and iucA are considered the most reliable molecular biomarkers for distinguishing hvKp from cKp. Although the presence of these five biomarkers does not completely capture the full hypervirulent phenotype owing to the multifactorial nature of *K. pneumoniae* pathogenesis, they serve as robust indicators of hypervirulence (Tang et al., 2025). Certain genetic elements can code for factors including increased capsule production-K1 and K2 capsule types, multiple siderophore systems, and the genotoxin colibactin, which are some of the unique features of hypervirulent *Klebsiella* strains. Additionally, hypermucoviscosity is considered one of the defining phenotypic characteristics of hvKp, further contributing to its enhanced virulence and invasive potential (Russo and Marr, 2019). An overview of major virulence factors of classical and hypervirulent *Klebsiella pneumoniae* involved in respiratory infection and dissemination has been depicted in Figure 1.

The figure provides a comparative overview of the major virulence determinants of classical (*cKp*) and hypervirulent (*hvKp*) *Klebsiella pneumoniae* involved in pathogenesis and systemic dissemination. Core virulence factors include type 1 and type 3 fimbriae, LPS, outer membrane proteins (*ompA, ompK35,* and *ompK36*), siderophore systems, and virulence-associated genes such as *uge, wabG,* and *rcsB*, which collectively contribute to adhesion, biofilm formation, capsule production, immune evasion, and bacterial persistence. In contrast, hvKp possesses additional hypervirulence-associated determinants carried by the pLVPK virulence plasmid, including *rmpA/rmpA2, iroB, iuc,* and *peg-344*, along with the *clb* gene cluster responsible for colibactin production and the regulatory genes *kvrA/kvrB*. Furthermore, siderophore-mediated iron sequestration promotes HIF-1α stabilization in respiratory epithelial cells, facilitating epithelial barrier disruption and bacterial dissemination into the bloodstream.

## 3 Exploring bacteriophage-based therapeutics for *Klebsiella pneumoniae* infections

The increasing prevalence of multidrug-resistant and hypervirulent *Klebsiella pneumoniae* has renewed interest in bacteriophage therapy as a precision antimicrobial strategy. Growing evidence from *in vitro*, preclinical, and clinical investigations demonstrates the therapeutic potential of lytic bacteriophages across diverse *K. pneumoniae* infections. A comprehensive summary of the available *in vitro* studies investigating the therapeutic efficacy of bacteriophages against *K. pneumoniae* is presented in Table 1. The following sections critically synthesize current evidence, emphasizing therapeutic outcomes, delivery strategies, and translational considerations that influence successful clinical application (Muraya et al., 2022; Bris et al., 2025; Kou et al., 2024). The following sections critically synthesize current evidence, emphasizing therapeutic outcomes, delivery strategies, and translational considerations that influence successful clinical application.

**Table 1:** *In vitro* evaluation of *Klebsiella* bacteriophages: methodologies and outcomes.

Targeted strain	Phage	Experimental focus	Outcome	Reference
*Klebsiella pneumoniae* B5055	KPO1K2	Intracellular phage delivery using liposomal encapsulation	Enhanced intracellular delivery with protection from neutralizing antibodies	Singla et al. (2016)
*Klebsiella pneumoniae* ST11-KL64	vB_KpnP-6 K2	Cytokine and chemokine expression profiling	Reduced IL-1β, TNF-α, CXCL1, CXCL2, CXCL3 expression, and enhanced IFN-β-associated CXCL10 signaling.	Pan et al. (2026)
*Klebsiella pneumoniae* KLEB009	vB_KpnP_KPN7 and vB_KpnM_KPN8	Cytokine profiling	Did not induce excessive TNF-*α* or IL-6 production.	Schubert et al. (2022)
*Klebsiella pneumoniae* KL23 clinical isolates	Dlv622, Seu621, FRZ284	Phage–antibiotic synergy	Levofloxacin–Seu621 combination exhibited strongest antibacterial activity	Gorodnichev et al. (2025)
Metallo-β- lactamase (MBL)-producing *K. pneumoniae*	vB_KpP_AttikonH1, vB_KpP_AttikonH2, vB_KpP_AttikonH4, vB_KpP_AttikonH5	Phage–antibiotic synergy	Synergistic interactions were observed with amikacin, meropenem, colistin, and ceftazidime/avibactam against most isolates	Paranos et al. (2025)
88 MDR *K. pneumoniae* (Kp) isolates	P28, P52, P60, P61, P67, P79, P85	Phage–antibiotic synergy	Phage–antibiotic combinations Enhanced antibacterial activity and reduced resistance emergence	Zhao et al. (2024)
*K. pneumoniae* 61	*K. pneumoniae* phage VB_KpP_HS106-endolysin Lys59	Phage endolysin evaluation	Lys59 produced 4-log reduction in *K. pneumoniae*	Zhang et al. (2026a)
*K. pneumoniae* 84	Kp84B Engineered endolysins CHU-1 and CHU-2	Phage endolysin evaluation	Exhibited significantly enhanced bactericidal activity	Chen et al. (2024)
*K. pneumoniae* IS43	Engineered Klebsiella phage endolysisn LysK1-Sub5	Phage endolysin evaluation	Improved antibacterial activity, expanded lytic spectrum	Gao et al. (2026)

### 3.1 Respiratory tract infections (RTI)

*Klebsiella pneumoniae* is a leading cause of community-acquired, hospital-acquired, and ventilator-associated pneumonia, with multidrug-resistant and carbapenem-resistant strains posing major therapeutic challenges (Li et al., 2025; Chen I. R. et al., 2022). Clinically, *K. pneumoniae* pneumonia is often associated with severe pulmonary manifestations such as extensive lung consolidation, pleural effusion, necrotic lesions, cavitation, and lung abscess formation (Jin et al., 2025). The pathogen accounted for approximately 176,000 LRI-associated deaths globally in 2021, highlighting its substantial public health burden and the urgent need for alternative therapeutic strategies (Li et al., 2025).

In response to the growing challenge of antibiotic resistance, several studies have demonstrated the therapeutic potential of bacteriophages against *K. pneumoniae*-induced respiratory infections. An environmentally isolated Siphoviridae phage was evaluated in a murine pneumonia model infected with NDM-producing *K. pneumoniae*. Administration of a phage suspension (3 × 10^9^ PFU/mL) resulted in complete survival of infected mice and the absence of pneumonia symptoms, demonstrating potent activity against highly antibiotic-resistant strains (VinodKumar et al., 2025). Similarly, intranasal treatment with the lytic phage VTCCBPA43 (2 × 10^9^ PFU/mouse) in a BALB/c mouse pneumonia model infected with *K. pneumoniae* MTCC109 significantly reduced pulmonary bacterial burden, alleviated lung lesions, and improved overall disease outcomes (Anand et al., 2020). Further evidence was provided by a study employing the Drexlerviridae phage PSKP16 against a *β*-lactamase-producing, K2 hypervirulent *K. pneumoniae* strain (KP12) in a murine intranasal pneumonia model. Phage administration at an MOI of 10 markedly reduced bacterial loads in the lungs, improved survival, mitigated lung pathology, decreased inflammatory cell infiltration, and inhibited biofilm formation (Rahimi et al., 2023) adding on to the findings demonstrating the effectiveness of phage therapy in controlling *K. pneumoniae*-associated respiratory infections and supporting its development as a promising alternative or adjunct to conventional antibiotic treatment.

### 3.2 Urinary tract infections (UTI)

As the second most common cause of uncomplicated urinary tract infections (UTIs), *Klebsiella pneumoniae* frequently infects the bladder, urethra, kidneys, and prostate, resulting in clinical manifestations ranging from urinary frequency and hematuria to severe systemic complications (Filev et al., 2025; Rizvi et al., 2025; Krawczyk et al., 2022; Jin et al., 2025). Several *in vivo* studies have provided compelling evidence supporting the efficacy of bacteriophage therapy against *K. pneumoniae*-induced urinary tract infections, demonstrating significant bacterial clearance and improved therapeutic outcomes. In a preclinical murine UTI model, a bacteriophage cocktail targeting the highly drug-resistant strain KpnBHU09 achieved complete eradication of infection following intravesical administration of either two doses of 1 × 10^5^ PFU/mouse or a single dose of 1 × 10^9^ PFU/mouse (Singh et al., 2024). Similarly, a six-phage cocktail comprising 311F, 05F, Centimanus, Storm, Anoxic, and SoFaint effectively controlled infection caused by the uropathogenic strain Top52 in female C57BL/6 J mice. Administration of 10^6^ PFU resulted in a rapid decline in bacterial burden within 4 h, with no detectable *Klebsiella* cells observed by days 6 and 7 (Jariah et al., 2025). Additional evidence was provided by studies evaluating phages vB_KpnP_K3-ULINTkp1 and vB_KpnP_K3-ULINTkp2 against the uropathogenic strain QAMH130326/0185 (ST13, K3) in a *Galleria mellonella* infection model. Phage treatment at MOI 10–100 significantly reduced bacterial burden and improved larval survival to 67% (Laforêt et al., 2022).

### 3.3 Bloodstream infections

*Klebsiella pneumoniae* is a major cause of bloodstream infections (BSIs) in both healthcare and community settings and is the second most common Gram-negative pathogen causing bacteremia after *Escherichia coli*. The emergence of hypervirulent, multidrug-resistant (MDR), and carbapenem-resistant lineages has substantially increased the clinical burden of BSIs, which frequently progress to septicemia or septic shock with high morbidity and mortality (Fostervold et al., 2024; Reid et al., 2019; Quispe-Villegas et al., 2025; Jin et al., 2025).

Several *in vivo* studies have demonstrated the therapeutic potential of bacteriophages against *K. pneumoniae*-associated bloodstream infections. In a murine septicemia model, a phage cocktail comprising ΦKpBHU4, ΦKpBHU7, and ΦKpBHU14 was evaluated against the MDR strain KpnBHU101. Intraperitoneal administration of a single dose of 1 × 10^5^ PFU/mouse protected animals from fatal septicemia and significantly improved survival, highlighting the efficacy of phage cocktails against drug-resistant bloodstream infections (Singh et al., 2022). Similarly, a C57BL/6 J mouse bacteremia model infected with the MDR ST258 strain demonstrated remarkable therapeutic benefits following treatment with the phages Pharr (P1) and ϕKpNIH-2 (P2). Administration at MOIs of 1 and 10 resulted in complete (100%) survival, while phage cocktail therapy also minimized the emergence of phage-resistant bacterial populations (Hesse et al., 2021). Further evidence was provided by studies utilizing phage vB_KpnP_Henu1_3 against the hypervirulent K1 serotype strain Kp1049 in a mouse peritonitis-sepsis model. Intraperitoneal treatment at MOIs ranging from 0.01 to 100 significantly enhanced survival across all treatment groups (Zhang et al., 2026b).

### 3.4 Skin and soft tissue infections

*Klebsiella pneumoniae* has emerged as an important cause of skin and soft tissue infections (SSTIs), including wound infections, burn wound infections, and necrotizing soft tissue infections. In burn patients, the organism can rapidly colonize wound surfaces and disseminate systemically, leading to invasive infections and septic complications. The increasing prevalence of multidrug-resistant (MDR) *K. pneumoniae* further complicates treatment and contributes to prolonged hospitalization, poor clinical outcomes, and increased mortality (Jin et al., 2025; Chen P. et al., 2025; Kelishomi et al., 2024; Wang et al., 2026).

Several *in vivo* studies have demonstrated the therapeutic potential of bacteriophages against *K. pneumoniae*-associated wound and skin infections. In a murine surgical wound infection model involving *K. pneumoniae* ST782, phage therapy produced a rapid and sustained reduction in bacterial burden, with wound cultures becoming negative by day 3. Bacterial counts remained significantly lower throughout the treatment period, and the phage-treated group exhibited the most favorable healing outcomes (Kelishomi et al., 2024). Similarly, the efficacy of bacteriophage ZCKP8 was evaluated against *K. pneumoniae* KP/15 in a rat wound infection model. Treatment using both phage suspension and phage-loaded gel formulations markedly enhanced wound healing, with wound closure reaching up to 99% by day 17 (Fayez et al., 2021). Additional evidence was provided by a study evaluating a three-phage cocktail comprising PH1, P24, and P39 against the carbapenem-resistant *K. pneumoniae* strain 140,156 (ST11-KL64) in a C57BL/6 murine wound infection model. Phage treatment significantly reduced wound bacterial burden and accelerated wound closure compared with untreated controls (Feng et al., 2024). Together, these findings demonstrate the potent antibacterial and wound-healing properties of bacteriophages and support their development as promising therapeutic agents for managing MDR *K. pneumoniae*-associated skin and soft tissue infections.

Beyond these infections, bacteriophage therapy has also demonstrated efficacy against *Klebsiella pneumoniae* in other disease models. In a murine having gastrointestinal colonization and renal infection model using carbapenemase-producing *K. pneumoniae* (KPC), a phage cocktail comprising Pharr, Soft, and Spivey significantly reduced renal bacterial burden, achieving approximately 100-fold and 1,000-fold reductions with 24-h and 12-h dosing intervals, respectively (Zagaliotis et al., 2024). In a murine mastitis model (ICR mice), a cocktail of CM_Kpn_HB132952, CM_Kpn_HB143742, and CM_Kpn_HB154724 targeting MDR *K. pneumoniae* KPHB132952 outperformed single-phage treatment in reducing bacterial counts (Liang et al., 2023). Additionally, in a gastrointestinal colonization model, combined administration of ϕK64-1 and ϕKO1-1 achieved long-term decolonization of K64 carbapenem-resistant *K. pneumoniae* in 52.3% of C57BL/6 mice for up to 56 days (Liu et al., 2022). These studies collectively highlight the broad applicability of bacteriophages in controlling bacterial burden, promoting recovery, and treating diverse *K. pneumoniae*-associated diseases.

Collectively, these *in vivo* studies demonstrate the therapeutic potential of bacteriophages against diverse *Klebsiella pneumoniae* infections across multiple disease models. However, variations in phage selection, dosing regimens, infection models, and routes of administration limit direct comparisons and hinder the establishment of standardized treatment strategies. Although phage cocktails consistently exhibited greater therapeutic efficacy and reduced the emergence of phage-resistant bacteria than monophage therapy, further comparative studies are required to validate these findings. Therefore, efforts should prioritize standardized preclinical protocols, comprehensive immune response evaluations, and well-designed clinical studies to facilitate the successful translation of phage therapy into routine clinical practices. Recent evidence of bacteriophage therapy against *Klebsiella pneumoniae* infection models has been depicted in Table 2.

**Table 2:** *In vivo* therapeutic studies of bacteriophages against *Klebsiella pneumoniae* infections.

Disease	In vivo model	Targeted strain	Phages	Delivery route	Outcome	References
Bronchoalveolar pneumonia	BALB/c mouse model	*K. pneumoniae* MTCC109	VTCCBPA43	Intransal	Reduced lung bacterial burden	Anand et al. (2020)
Acute pneumonia	BALB/c mouse model	β-lactamase-producing, K2 hypervirulent *K. pneumoniae*	PSKP16	Intransal	Reduced lung bacterial burden	Rahimi et al. (2023)
Pneumonia	Female mice model	NDM-4 producing *Klebsiella pneumoniae*	*Klebsiella* phage (Siphoviridae)	intraperitoneal	100% survival and complete clearance of bacterial burden	VinodKumar et al. (2025)
Acute pneumonia	PK2420	Hypervirulent *Klebsiella pneumoniae* K2420	C57BL/6 J mice model	Intratracheal administration	100% survival, with reduction in lung bacterial burden and suppression of IL-1β, IL-6, and TNF-α,	Chen J. et al. (2025)
UTI	Female C57BL/6 J mice model	ESBL-producing *Klebsiella pneumoniae* Top52	311F,05F, Anoxic, SoFaint, Centimanus, Storm	Transurethral	Reduced bacterial burden in urine	Jariah et al. (2025)
UTI	Female Swiss albino mice model	*Klebsiella pneumoniae* KpnBHU09	KpnBHU1, KpnBHU2, KpnBHU3	Transurethral, rectal, oral, and subcutaneous	Transurethral delivery achieved complete eradication of UTI	Singh et al. (2024)
UTI	Galleria mellonella model	ST13 K3 *Klebsiella pneumoniae*	vB_KpnP_K3-ULINTkp1 and vB_KpnP_K3-ULINTkp2	Larval injection	improved larval survival and reduced bacterial burden;	Laforêt et al. (2022)
Bacteremia	C57BL/6 J mice model	*Klebsiella pneumoniae* (ST258)	Pharr (P1) and ϕKpNIH-2 (P2)	Intraperitoneal (IP)	Phage cocktail treatment achieved 100% survival in mice	Hesse et al. (2021)
Septicemia	Swiss albino mouse	*Klebsiella pneumoniae* (KpnBHU101)	ΦKpBHU4, ΦKpBHU7, and ΦKpBHU14	Intraperitoneal (IP)	Phage cocktail provided 100% survival with reduced bacterial burden.	Singh et al. (2022)
Bacteremia	Mouse model	K2 hypervirulent *Klebsiella pneumoniae* FK1979 and φFK1979-resistant mutant (R3)	φFK1979 and phiR3	Intraperitoneal (i.p.)	Improved survival	Tang et al. (2024)
Peritonitis, sepsis	C57BL/6 mouse	*K. pneumoniae* Kp1049	vB_KpnP_Henu1_3	Intraperitoneal (IP)	Improved survival and reduced bacterial loads.	Zhang et al. (2026b)
Wound infection	Rat model	MDR *Klebsiella pneumoniae* KP/15	ZCKP8	Topical application	Accelerated wound healing and reduced bacterial infection.	Fayez et al. (2021)
Surgical wound infection	BALB/c mouse model	*K. pneumoniae* ST782	PSKP16	Topical application	Accelerated wound healing and reduced bacterial burden.	Kelishomi et al. (2024)
wound infection	C57BL/6 murine model	ST11-KL64 carbapenem-resistant *Klebsiella pneumoniae* strain	PH1, P24, and P39	Topical application	Reduced wound bacterial burden and increased wound healing	Feng et al. (2024)
Gastrointestinal colonization and renal infection	Neutropenic CF-1 mouse model	ST258 KPC-producing *K. pneumoniae* clinical isolate 39,427	Pharr, Soft, and Spivey	Intraperitoneal	Reduced bacterial burden	Zagaliotis et al. (2024)
Mastitis	ICR mice	K57 *K. pneumoniae* strain	CM_Kpn_HB132952, CM_Kpn_HB143742 and CM_Kpn_HB154724	Intramammary	Significantly reduced bacterial burden	Liang et al. (2023)
Intestinal decolonization	C57BL/6 mouse model	K64 carbapenem resistant *K. pneumoniae* strain	ϕK64-1 and ϕKO1-1	Oral + intraperitoneal administration	Achieved long-term decolonization in 52.3% of mice	Liu et al. (2022)

### 3.5 Clinical evidence and translational progress

Despite the evidence from the *in vitro* and *in vivo* studies, the clinical application of bacteriophage therapy against *Klebsiella pneumoniae* infections is still evolving. Current clinical evidence available consists of compassionate-use case reports as a last resort of medicine for *Klebsiella pneumoniae* infections, especially against MDR strains of *Klebsiella pneumoniae* (Kou et al., 2024; Gholizadeh et al., 2024). Certain evidence has shown successful clinical outcomes by either giving bacteriophages alone or with antibiotics, showing an efficient treatment methodology where conventional antibiotics failed (Kou et al., 2024; Marino et al., 2025).

Clinical studies of *Klebsiella pneumoniae* bacteriophages have been reported against different infections, including pneumonia, UTIs, and prosthetic joint infections (PJIs). In one study, the lytic *Klebsiella pneumoniae* phage KpJH46Φ2 was used against the *K. pneumoniae* complex strain KpJH46 as part of compassionate use in a patient with chronic biofilm associated prosthetic knee infection. The bacteriophage was administered intravenously in combination with long term minocycline. The study demonstrated the resolution of local signs and symptoms, with the patient recovering from the joint infection without any adverse events and remaining asymptomatic for 34 weeks after completion of therapy, highlighting the therapeutic potential of *Klebsiella pneumoniae* phage therapy in combination with antibiotics for device-associated biofilm infections (Cano et al., 2021).

Similarly, another study on the use of *Klebsiella pneumoniae* phages was conducted in a UTI patient who was a kidney transplant recipient. The study focused on the use of a three-phage cocktail consisting of Metamorpho, Mineola, and pKp20 for the treatment of infections caused by extended-spectrum *β*-lactamase (ESBL) producing *K. pneumoniae*. The phages were administered intravenously twice a day for 28 days without any concomitant antibiotics. The treatment was successful, with no recurrence of ESBL producing strains during one year of follow-up, and the subsequent isolates remained susceptible to oral antibiotics, highlighting the potential of phage monotherapy against *K. pneumoniae*-associated UTIs (Le et al., 2023).

*Klebsiella pneumoniae* bacteriophages have demonstrated their translational efficacy in patients with ventilator-associated pneumonia (VAP), where patients received a lytic phage cocktail targeting MDR *K. pneumoniae* via nebulization twice daily for 14 days along with standard antibiotic therapy. Targeted phage therapy achieved microbiological eradication of *K. pneumoniae* in 86% of the patients, leading to earlier discontinuation of antibiotic therapy and ventilatory support, thereby further demonstrating the clinical application of bacteriophages as a promising treatment strategy against *Klebsiella pneumoniae* infections from a translational perspective (Gorodnichev et al., 2026). Further clinical studies on bacteriophage therapy against *Klebsiella pneumoniae* infections are summarized in Table 3.

**Table 3:** Clinical studies investigating phage delivery approaches against *Klebsiella pneumoniae* infections.

Disease	Delivery strategy	Human population	Phages	Targeted strain	Study analysis	Reference
VAP	Aerosolized delivery via vibrating-mesh nebulizer	9-month-old infant in ICU	Kp240, Kp67 (targeting CRKP) Ab35 (targeting CRAB)	Carbapenem-resistant *Klebsiella pneumoniae* (CRKP; KL25/ST11) and *Acinetobacter baumannii* (XDR-AB; KL14/ST25)	-Rapid microbiological clearance supported by personalized phage therapy for MDR respiratory infections. -Demonstrated the translational potential of aerosolized precision phage therapy.	Yan et al. (2025)
VAP	Nebulizer	Adult ICU patients	Seven-phage cocktail	MDR *K. pneumoniae*	-Rapid bacterial clearance supported by personalized phage therapy. -Nebulized delivery facilitated precision phage therapy for MDR pneumonia.	Gorodnichev et al. (2026)
MDR pulmonary infection	Nebulizer	54-year-old male patient	FKp_GWPB35, FKp_GWPA139	MDR *K. pneumoniae* Kp7450	-Nebulized personalized phage therapy improved pulmonary infection despite emergence of phage-resistant *K. pneumoniae*. -Phage resistance reduced bacterial virulence, maintaining clinical benefit.	Li et al. (2023)
UTI	Oral administration and intravesical bladder irrigation	58-year-old renal transplant recipient	Personalized anti-*Klebsiella pneumoniae* bacteriophage	ESBL-producing *Klebsiella pneumoniae*	-Resolved recurrent ESBL-*K. pneumoniae* UTI following repeated antibiotic failure. -Oral and intravesical phage delivery supported localized adjunctive therapy for persistent infection.	Kuipers et al. (2019)
Recurrent UTI	IV	70-year-old female kidney–liver transplant recipient	Metamorpho, Mineola, pKp20	ESBL-producing *K. pneumoniae*	-Demonstrated the feasibility of intravenous phage monotherapy for recurrent ESBL-*K. pneumoniae* UTI. -Reduced recurrence supporting targeted systemic phage therapy.	Le et al. (2023)
Prosthetic joint infection (PJI)	IV	62-year-old male	KpJH46Φ2	*K. pneumoniae* complex (KpJH46)	-Demonstrated the feasibility of intravenous personalized monophage therapy for eradicating biofilm-associated prosthetic joint infection. -Supports systemic phage delivery as a potential alternative when surgery is not feasible.	Cano et al. (2021)
PJI with septicemia	Catheter-mediated phage irrigation	60-year-old female	ΦKpnBHU1, ΦKpnBHU2, ΦKpnBHU3	Pan-drug-resistant *K. pneumoniae*	-Localized phage irrigation rapidly controlled septicemia and reduced bacterial burden. -Prolonged local catheter-based phage delivery supported management of chronic implant-associated biofilm infection.	Nath (2025)
PJI	intra-articular (IA) and IV	70-year-old male	KP1, KP2	ESBL-producing *K. pneumoniae*	-Combined intra-articular and intravenous phage delivery enabled targeted management of recalcitrant prosthetic joint infection. -Supports personalized multimodal phage administration for prosthetic joint infections.	Doub et al. (2022)

Although these encouraging clinical experiences represent an important transition from preclinical findings to clinical application, the current evidence is derived predominantly from compassionate-use cases and small uncontrolled studies, exhibiting considerable differences in phage selection, formulation, dosing regimens, routes of administration, and outcome assessment (Marino et al., 2025). Due to these limitations, there is a need for standardized phage manufacturing, harmonized regulatory frameworks, and adequately powered randomized controlled trials (RCTs) to validate the safety, efficacy, and optimal clinical application of bacteriophage therapy against *K. pneumoniae* infections.

## 4 The immunological paradigm of bacteriophage therapy

### 4.1 Targeting intracellular *Klebsiella pneumoniae*: host–phage interactions and therapeutic considerations

Recent studies have revealed that *Klebsiella pneumoniae*, particularly hypervirulent strains, can survive intracellularly following phagocytosis by macrophages through inhibition of lysosome fusion with the *Klebsiella* containing vacuole (KCV), thereby establishing intracellular reservoirs of infection. This intracellular lifestyle contributes to persistence, metastatic dissemination, and tolerance to antibiotics with poor cellular penetration, such as aminoglycosides, highlighting the need for novel therapeutic strategies against persistent intracellular infections (Jiang et al., 2018). Although bacteriophages have demonstrated considerable efficacy against extracellular pathogens, intracellular localization of *K. pneumoniae* remains a major therapeutic challenge because it limits direct phage–bacterium interactions and exposes phages to immune-mediated clearance mechanisms, including phagocytic uptake and neutralizing antibodies (Fajardo-Lubian and Venturini, 2023; Bichet et al., 2021).

**Figure 2:**
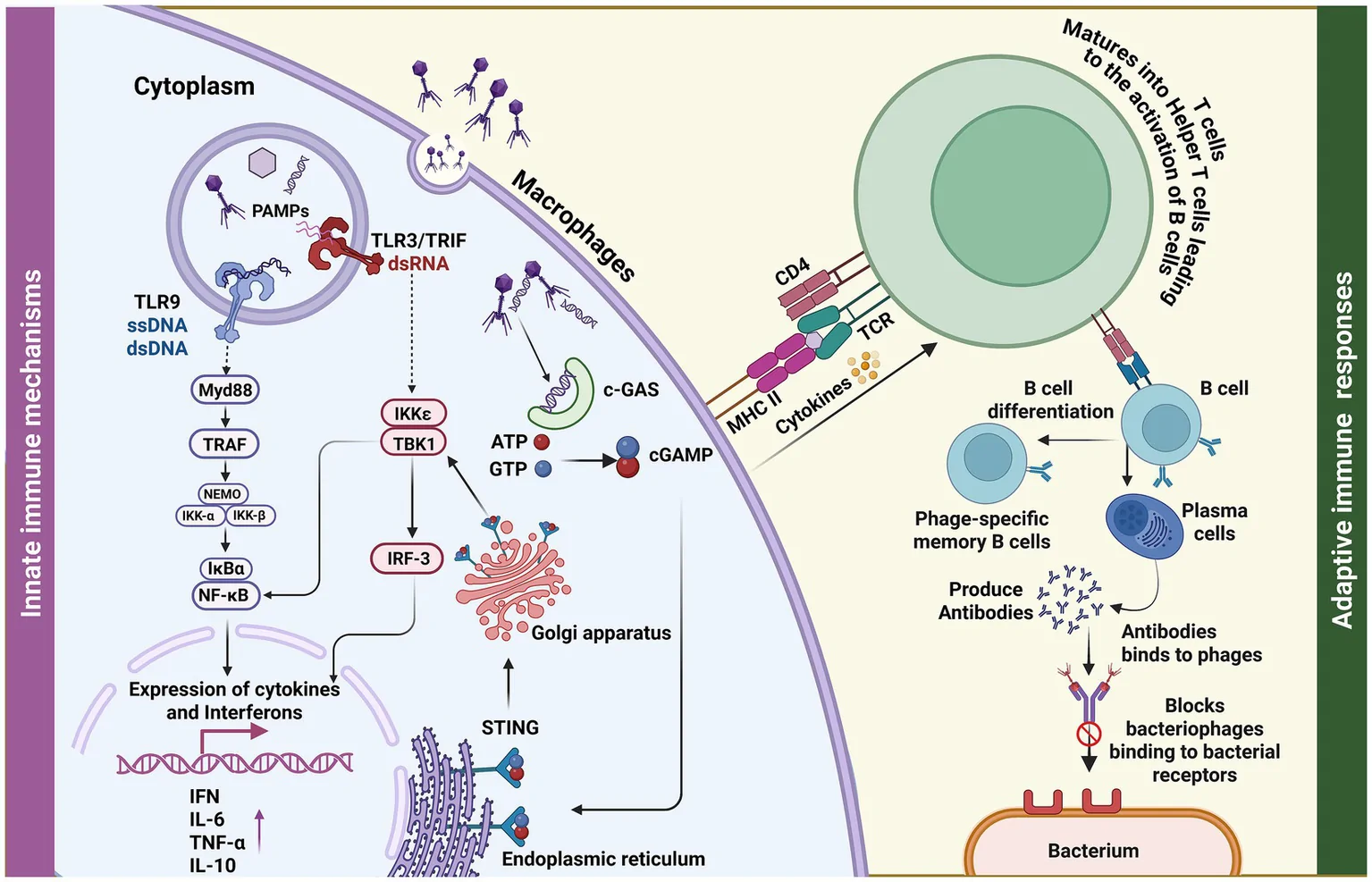
Innate and adaptive immune responses to bacteriophage therapy.

Recent evidence has demonstrated that bacteriophages can enter mammalian cells through macropinocytosis and remain biologically active following internalization. Cellular uptake has been observed in epithelial, endothelial, fibroblast, and macrophage cell lines, with internalized phages accumulating intracellularly without inducing major inflammatory responses. These findings suggest that phages possess the potential to access intracellular bacterial reservoirs and may influence phage biodistribution, persistence, and therapeutic outcomes (Bichet et al., 2021, 2023). In parallel, therapeutic phages interact with both innate and adaptive immune systems, generating immune responses that influence phage pharmacokinetics, persistence, and clearance. Notably, antibody-mediated phage elimination may either reduce therapeutic efficacy through premature phage clearance or facilitate removal of residual phages after bacterial eradication, depending on the timing of the immune response (Démoulins et al., 2025; Shan et al., 2018). The interplay between bacteriophages and the host immune system, encompassing both innate and adaptive components, is illustrated in Figure 2.

Together, the available evidence indicates that bacteriophages possess the ability to access intracellular compartments, providing a promising strategy for targeting intracellular *Klebsiella pneumoniae*. The therapeutic success of this approach is influenced by multiple factors, including the efficiency of phage internalization, intracellular persistence, and the dynamic interactions between bacteriophages and the host immune system. A comprehensive understanding of these mechanisms is essential for optimizing bacteriophage-based interventions against intracellular infections, including those caused by *K. pneumoniae*.

### 4.2 Phage recognition by the host immune system

Bacteriophages are recognized by host pattern-recognition receptors (PRRs), including Toll-like receptors (TLR2, TLR4, TLR7, TLR8, and TLR9), RIG-I-like receptors, and other viral sensing pathways, initiating innate immune responses that contribute to the immunomodulatory effects of phage therapy (Kapoor et al., 2025; Muema et al., 2025). Experimental studies indicate that phage therapy generally exerts anti-inflammatory effects *in vivo*, primarily through efficient bacterial clearance, whereas direct exposure of immune cells to purified phages *in vitro* may activate pro-inflammatory signaling through receptors such as TLRs and the cGAS–STING pathway (Somsri et al., 2026).

Recent evidence suggests that phage recognition is influenced by both phage-specific characteristics and preparation quality. For example, incomplete activation of TLR9 has been reported, while residual bacterial components, including peptidoglycan, lipoproteins, and bacterial DNA, may confound immune responses by activating additional PRRs despite endotoxin removal (Bosco et al., 2026). Mammalian cells can identify phage particles also through other viral particle-specific receptors such as C-type lectin domain family 4 and human tyrosine protein kinase receptor UFO (encoded by the *axl* gene) that can induce the signal through several transcription factors, including Janus kinase-signal transducer and activator of transcription, mitogen-activated protein kinase, Salvador-Warts-Hippo (Hippo), and phosphatidylinositol 3-kinase/protein kinase B (Somsri et al., 2026). Following phage recognition, activation of host sensing pathways can trigger immune responses such as endocytosis, phagocytosis, and interferon production. Depending on the downstream signaling cascades and transcriptional regulators involved, including nuclear factor-kappa B (NF-κB) and MiT family transcription factors, these responses may result in either pro-inflammatory or anti-inflammatory outcomes (Kasprzak et al., 2025; Somsri et al., 2026).

### 4.3 Phagocytic recognition and uptake of bacteriophages

The mononuclear phagocyte system (MPS) comprising phagocytic cells plays a pivotal role in the clearance of foreign particles, including phages from the circulatory pathways (Le et al., 2025; Bosco et al., 2026). Recognition of phages by these cells is influenced by phage surface characteristics, which affect phagocytic uptake, intracellular trafficking, and degradation through the endolysosomal pathway, ultimately influencing phage persistence and bioavailability (Nguyen and Yates, 2021; Le et al., 2025).

In respiratory tract infections, alveolar macrophages are the first immune cells to encounter inhaled bacteriophages. Besides contributing to pathogen clearance, they regulate pulmonary inflammation and tissue repair, suggesting that macrophage–phage interactions may significantly influence the persistence, distribution, and therapeutic efficacy of inhaled phage therapy (Zborowsky et al., 2025).

### 4.4 Bacteriophage-mediated cytokine responses

Recognition of bacteriophage particles by host pattern-recognition receptors (PRRs) initiates intracellular signaling cascades that modulate cytokine production and shape downstream immune responses, forming a central mechanism of cytokine regulation in phage therapy (Podlacha et al., 2024; Cianci et al., 2025). Characterization studies have shown two distinct immune pathways: a vacuolar response that is entirely Myeloid differentiation primary response (MyD) 88-dependent and drives expression of pro-inflammatory cytokines such as Interleukin (IL)1*α*, IL1*β*, and tumor necrosis factor (TNF), and a cytosolic response that is Interferon Regulatory Factor 3 (IRF3)-dependent and activates a distinct set of 106 genes, including Type I interferons, such as IFNβ. Phages can also stimulate IFN responses. Both type I interferons (IFN-α and IFN-β), which play roles in antigen presentation and suppression of macrophage activity, and type II interferons (IFN-*γ*), which drive Th1-mediated inflammation, may be induced by phage exposure.

Exposure to phages can induce various immune responses depending on their genome. Peripheral blood mononuclear cells (PBMCs) getting exposed to double-stranded DNA (dsDNA) phages can produce pro-inflammatory cytokines (TNF, IL-1α, IL-1β, and IL-6), along with IL-10. On the other hand, double-stranded RNA (dsRNA) phages can elicit the production of IL-6, IFN-β, and CCL4.

Experimental evidence indicates that these responses are highly phage dependent. For example, lung epithelial cells exposed to podovirus PEV2 exhibited increased IL-6 and TNF production, whereas siphovirus DMS3 induced minimal cytokine responses (Cianci et al., 2025). In contrast, *in vivo* studies suggest that successful phage therapy predominantly attenuates inflammation as bacterial burden declines. In murine *Klebsiella pneumoniae* pneumonia, intranasal phage cocktail treatment significantly reduced IL-1β, TNF-α, and IL-6 expression while improving bacterial clearance and survival (Ding et al., 2026). Similarly, phage 6 K2 reduced IL-1β, TNF-α, and neutrophil-recruiting chemokines (CXCL1, CXCL2, and CXCL3) in *K. pneumoniae*-infected THP-1 cells, indicating reduced inflammatory cell recruitment and tissue damage (Pan et al., 2026).

### 4.5 Bacteriophage-induced adaptive immune responses

Phages normally enter epithelial cells via endocytosis or transcytosis without the activation of traditional MHC-I restricted antiviral responses. Due to the same, certain transitions to phage-mediated adaptive immunity lack the cytotoxic CD8 + T-cell activation. Instead, the adaptive arm regarding phage administration is dominated by CD4 + T-cell-mediated humoral immunity. Initial exposure typically elicits a primary immunoglobulin M (IgM) response, which matures into a more persistent immunoglobulin G (IgG) profile upon repeated administration (Bosco et al., 2026).

Both preclinical and clinical studies have demonstrated the generation of anti-phage antibodies following therapeutic phage administration in murine models and human subjects. These antibodies can facilitate phage clearance from the circulation and, in some cases, neutralize phage infectivity against target bacteria, thereby influencing therapeutic efficacy. The antibody response is typically polyclonal, which can target multiple phage structural components, including major capsid proteins, receptor-binding proteins, and accessory decorative proteins such as Hoc and Soc. Notably, phage-neutralizing antibodies have also been detected in up to 40% of individuals with no prior exposure to phage therapy (Bernabéu-Gimeno et al., 2024; Le et al., 2025).

Although prolonged and repeated administration of bacteriophages from a therapeutic perspective may induce the production of anti-phage antibodies capable of promoting phage clearance, several strategies have been proposed to minimize the impact of such immune responses on the therapeutic efficacy of phage therapy. Selection, screening, and characterization of therapeutic phages are essential, as individual phages differ in their immunogenicity, with some eliciting minimal host immune activation while others induce robust innate and adaptive immune responses. Therefore, preclinical evaluation of host–phage immune interactions should focus on identifying suitable therapeutic candidates with lower immunogenic potential (Démoulins et al., 2025; Bosco et al., 2026). Furthermore, optimization of the route of administration and minimizing systemic exposure may help reduce immune-mediated phage clearance. Delivery strategies such as liposomal encapsulation, hydrogels, polymeric nanoparticles, spray-dried powders, and other protective formulations can shield phages from immune recognition, thereby enhancing phage stability, prolonging retention time, and improving targeted delivery (Cui et al., 2024; Bolsan et al., 2024). A recent clinical case report of nebulized monophage therapy in cystic fibrosis patients with chronic *Pseudomonas aeruginosa* and *Staphylococcus aureus* lung infections provided the first evidence of anti-phage neutralizing antibody generation following inhalational phage administration. Neutralizing antibodies were detected 10–42 days after treatment and persisted during follow-up; however, phage therapy remained safe, reduced bacterial burden, and improved clinical outcomes, indicating that antibody generation does not necessarily compromise therapeutic efficacy. These findings underscore the importance of longitudinal immune monitoring and personalized phage selection during repeated treatment courses (Bernabéu-Gimeno et al., 2024).

Collectively, these findings highlight that the clinical success of bacteriophage therapy depends on balancing the beneficial and adverse effects of antibody-mediated phage clearance. While premature neutralization of therapeutic phages may compromise bacterial killing, delayed clearance following successful bacterial eradication may facilitate the removal of residual circulating phages after they have fulfilled their antibacterial function (Démoulins et al., 2025). Therefore, personalized optimization of phage selection, dosing schedules, treatment duration, route of administration, and longitudinal immune monitoring should become integral components of clinical phage therapy to maximize therapeutic efficacy while minimizing undesirable immune-mediated phage clearance (Bosco et al., 2026).

### 4.6 Innate immune synergy in phage therapy

Beyond limiting the factors for minimizing antibody-mediated phage clearance, evidence also puts forward that certain host immune interactions contribute towards the therapeutic efficacy of bacteriophage therapy. The combination of phage-mediated bacterial lysis, thereby reducing the bacterial burden, and the elimination of residual bacteria and emergent phage resistant variants by innate immune cells establishes a synergistic interaction between bacteriophages and the host immune system rather than functioning as independent antibacterial agents (Van Belleghem et al., 2018). This concept of immune-phage synergy demonstrated by Roach et al., also emphasized that successful phage therapy required functional innate immunity, particularly the action of neutrophils, for complete bacterial clearance of the infecting pathogen (Roach et al., 2017). Similar synergy can also be found in this study regarding the action of capsule-targeting phages against hypervirulent *Klebsiella pneumoniae* infection, where the phage-resistant mutants generated as part of the therapy were eliminated by host immune mechanisms, resulting in superior therapeutic outcomes in immunocompetent hosts, whereas immunocompromised hosts required sequential administration of phages targeting resistant bacteria to achieve comparable therapeutic efficacy (Tang et al., 2024). These findings collectively demonstrate that, even though immune responses may promote phage clearance through antibody production, host immunity simultaneously acts as a critical partner in bacterial eradication, emphasizing that successful phage therapy relies on coordinated synergy between bacteriophages and the host immune systems (Van Belleghem et al., 2018; Bris et al., 2025).

Following internalization by macrophages and other antigen-presenting cells, phage-derived nucleic acids are recognized by endosomal Toll-like receptors (TLRs) and cytosolic sensors such as the cGAS–STING pathway. These signaling cascades activate NF-κB, IRF3, and other downstream mediators, leading to the production of cytokines and interferons that regulate inflammatory responses. Concurrently, phage antigens are processed and presented to CD4^+^ T cells, promoting B-cell activation and differentiation into plasma cells and memory B cells. The resulting anti-phage antibodies can neutralize bacteriophages, potentially influencing phage persistence, infectivity, and therapeutic efficacy.

## 5 Synergistic modalities in phage-based antimicrobial therapy

To improve therapeutic efficacy against multidrug-resistant *Klebsiella pneumoniae*, increasing attention has been directed toward combination strategies that integrate bacteriophages with complementary antimicrobial approaches. Current evidence supports the use of phage–antibiotic combinations, phage-derived enzymes, biofilm-targeting agents, and immunomodulatory strategies to enhance bacterial clearance, suppress resistance development, and improve treatment outcomes.

Among these approaches, phage–antibiotic synergy (PAS) is the most extensively investigated. PAS seeks to improve the treatment of multidrug-resistant infections while simultaneously limiting the emergence of phage-resistant bacterial populations. The effectiveness of PAS is influenced by the antibiotic class employed, as certain antibiotics can enhance phage activity through distinct mechanisms (Domingo-Calap et al., 2024; Supina and Dennis, 2025). Evidence for PAS has been demonstrated in a murine infection model involving the hypervirulent carbapenem-resistant *K. pneumoniae* ST11 strain Kp2058. Treatment with a seven-phage cocktail in combination with colistin produced significantly greater reductions in bacterial burden than either phage or antibiotic monotherapy, with similar effects observed across multiple organs, including the lungs, kidneys, spleen, and cecum (Zhao et al., 2024). Clinical evidence has also been reported in a patient with a chronic fracture-related infection caused by pandrug-resistant *K. pneumoniae* Kp36336, where a pre-adapted lytic myophage (vB_KpnM_M1) combined with meropenem, colistin, and subsequently ceftazidime/avibactam resulted in bacterial eradication, wound healing, fracture consolidation, and long-term infection resolution (Eskenazi et al., 2022). Mechanistically, PAS can enhance antibacterial efficacy by increasing phage adsorption and improving antibiotic access to bacterial targets. In addition, phage-driven selection of resistant mutants may restore antibiotic susceptibility. For instance, mutations in the *wcaJ* gene that confer phage resistance in *K. pneumoniae* have been associated with a 32-fold increase in susceptibility to ceftazidime. Collectively, these findings highlight PAS as a promising strategy for improving therapeutic efficacy while mitigating resistance in *K. pneumoniae* infections (Ding et al., 2025).

Although PAS has shown considerable promise in enhancing antibacterial efficacy, suppressing the emergence of phage-resistant bacterial mutants, and improving therapeutic outcomes, these interactions are not universally synergistic. The success of PAS depends on several factors, including the antibiotic mechanism of action, bacterial physiological state, treatment stoichiometry, phage characteristics, and the host microenvironment (Gu Liu et al., 2020; Kunz Coyne et al., 2024).

In particular, combining lytic bacteriophages with bacteriostatic antibiotics may result in antagonistic rather than synergistic effects. By inhibiting bacterial transcription or protein synthesis, bacteriostatic antibiotics reduce bacterial metabolic activity, thereby impairing phage genome replication and progeny production, ultimately diminishing antibacterial efficacy (Kunz Coyne et al., 2024). Moreover, bacteriostatic antibiotics such as chloramphenicol, tetracycline, erythromycin, and trimethoprim have been shown to delay phage development and promote CRISPR-mediated spacer acquisition, facilitating bacterial anti-phage immunity and emphasizing the importance of rational antibiotic selection during PAS (Dimitriu et al., 2022; Chen Q. et al., 2022).

Conversely, bactericidal antibiotics, particularly *β*-lactams and other cell wall-active agents, generally exhibit greater compatibility with lytic phages by maintaining bacterial metabolic activity while promoting complementary antibacterial mechanisms, making them more suitable candidates for PAS-based therapeutic strategies (Kunz Coyne et al., 2024).

Phage-derived enzymes have emerged as promising adjuncts to conventional antimicrobial therapies due to their targeted antibacterial activity and favorable safety profile. Unlike whole bacteriophages, these enzymes do not replicate within the host, providing greater predictability and facilitating regulatory approval. Among the diverse classes of phage enzymes, endolysins and depolymerases have attracted considerable attention for the treatment of *Klebsiella pneumoniae* infections (Yang et al., 2025; Bris et al., 2025). Endolysins are peptidoglycan hydrolases that degrade the bacterial cell wall, resulting in rapid bacterial lysis. Their ability to target highly conserved peptidoglycan structures makes the development of bacterial resistance relatively uncommon. Recent *in vitro* and *in vivo* studies have demonstrated the antibacterial efficacy of several lysins against multidrug-resistant *K. pneumoniae* strains. Furthermore, lysins can act synergistically with antibiotics and antimicrobials. Peptides, enhancing bacterial killing and improving treatment outcomes against resistant infections. Several studies have investigated the therapeutic potential of bacteriophage-derived endolysins against *Klebsiella pneumoniae*. One study characterized the phage-derived endolysin Lys59 from *K. pneumoniae* phage VB_KpP_HS106, which demonstrated potent antibacterial activity against multidrug-resistant *K. pneumoniae*, broad-spectrum activity against both Gram-positive and Gram-negative bacteria, high physicochemical stability, and effective antibiofilm activity (Zhang et al., 2026a). Recent studies have also explored synergistic strategies involving phage-derived endolysins. The *K. pneumoniae* phage endolysin LysKp84B was engineered with the membrane-penetrating peptides ApoE23 and COG133 to generate the variants CHU-1 and CHU-2, both of which exhibited significantly enhanced bactericidal activity against *K. pneumoniae*, *Acinetobacter baumannii*, *Enterococcus faecalis*, and *Staphylococcus aureus* compared with the native endolysin (Chen et al., 2024). Similarly, fusion of the *K. pneumoniae* phage-derived endolysin LysK1 with the antimicrobial peptide Sub5 (LysK1-Sub5) resulted in enhanced antibacterial activity, a broader lytic spectrum, and reduced bacterial burden with accelerated wound healing in a murine infection model, collectively highlighting the therapeutic potential of antimicrobial peptide–endolysin fusion strategies (Gao et al., 2026).

Depolymerases represent another important class of phage-derived enzymes with specific relevance to *K. pneumoniae*. These enzymes selectively degrade extracellular polysaccharide structures, including capsular polysaccharides (CPS), lipopolysaccharides (LPS), and biofilm-associated matrices. Due to their high substrate specificity, these enzymes effectively disrupt biofilms formed by *Klebsiella* and remove protective capsules, thereby enhancing bacterial susceptibility to antibiotics and host immune responses (Bris et al., 2025).

Overall, the available evidence indicates that synergistic modalities, particularly the combination of bacteriophages with antibiotics and the use of phage-derived enzymes, enhance antibacterial efficacy beyond that achieved with individual therapies. While phage–antibiotic combinations primarily improve bacterial killing and reduce the emergence of phage-resistant mutants, phage-derived enzymes and their synergistic combinations target bacterial cell walls, capsules, and biofilms through distinct mechanisms. The expanding range of these synergistic approaches provides multiple therapeutic options to address the heterogeneous nature of *Klebsiella pneumoniae* infections. Tailoring these interventions according to bacterial characteristics and the site of infection may further maximize the therapeutic potential of phage-based antimicrobial therapy.

## 6 Therapeutic phage delivery strategies

The successful implementation of bacteriophage therapy against *Klebsiella* infections or any other infections depends not only on the selection of effective phages but also on the development of delivery systems capable of preserving phage viability and ensuring efficient transport to the site of infection. Various administration routes, including oral, parenteral, topical, and inhalational delivery, have been investigated for therapeutic applications. Oral administration is a convenient and non-invasive approach widely used for gastrointestinal infections; however, phage survival is often compromised by the acidic gastric environment and absorption throughout the gastrointestinal tract. Parenteral administration, including intravenous, intramuscular, and subcutaneous routes, provides rapid systemic distribution and high bioavailability, making it suitable for severe infections. Topical administration has been successfully applied for wound, burn, ulcer, and prosthetic joint infections, where localized delivery allows direct targeting of infected tissues (Vila et al., 2024).

To enhance therapeutic performance, a wide range of formulation strategies have been developed to improve phage stability, bioavailability, and controlled release. Biomaterial-based platforms, particularly hydrogels, have shown considerable promise for localized wound therapy by improving phage retention, reducing biofilm formation, and enabling sustained release (Vila et al., 2024; Rotman et al., 2020; Bolsan et al., 2024; Loh et al., 2021). Similarly, nanocarrier systems, including liposomes, transferosomes, niosomes, and nanoparticles, protect phages from degradation and immune-mediated clearance while improving tissue penetration, intracellular delivery, and biofilm access. Among these, liposome-encapsulated phages have demonstrated enhanced macrophage uptake and intracellular localization, highlighting their potential for targeting persistent intracellular bacterial reservoirs (Loh et al., 2021; Wang et al., 2021; Pinto et al., 2021; Selim et al., 2025). For respiratory tract infections, aerosol-based delivery remains the most extensively studied approach. Nebulization generates aerosolized droplets capable of transporting phages directly to the lungs, enabling efficient deposition at the infection site. Both jet and vibrating mesh nebulizers have demonstrated effective pulmonary delivery, although phage stability can be affected by shear stress, humidity, and temperature (Wang et al., 2021; Selim et al., 2025). To address these limitations, dry powder inhalation formulations have been developed using techniques such as spray drying, lyophilization, and supercritical fluid spray drying. These formulations offer improved storage stability, enhanced deep-lung deposition, portability, and patient compliance. Emerging technologies, including microfluidic emulsification, also enable the production of highly uniform phage-containing microcapsules with controlled size and morphology (Encinas-Basurto et al., 2025; Vila et al., 2024; Bolsan et al., 2024).

The available evidence regarding bacteriophage delivery strategies, particularly from preclinical and emerging clinical studies, indicates that therapeutic success depends not only on the selection of effective phages but also on optimizing the delivery approach according to the site and nature of the infection. Beyond conventional routes of administration, advanced delivery platforms, including biomaterial-based systems, nanocarriers, and inhalable formulations, offer additional advantages such as improved phage stability, targeted delivery, and enhanced tissue penetration. The choice of an appropriate delivery system should therefore be guided by both the characteristics of the infecting pathogen and the physiological environment of the infected tissue. Integrating optimized delivery platforms with carefully selected bacteriophages has the potential to substantially improve the clinical efficacy of phage therapy against *Klebsiella pneumoniae* infections. Studies that have investigated phage delivery strategies against *Klebsiella pneumoniae* infections in human populations, and their key findings are summarized in Table 3.

## 7 Advancements in AI and computational phage research

The clinical implementation of bacteriophage therapy is constrained by challenges such as rapid identification of suitable therapeutic phages, prediction of phage–host compatibility, optimization of treatment regimens, and timely clinical deployment. Recent advances in artificial intelligence (AI), machine learning, and computational genomics have substantially accelerated these processes by improving phage discovery, characterization, and therapeutic selection using large-scale genomic and metagenomic datasets (Rahimian, 2025; de Paula Silva et al., 2025).

AI-based approaches have demonstrated particular value in predicting phage–host interactions, enabling strain-level identification of therapeutic phages directly from bacterial genomic information and phage–bacteria infection networks. These approaches have been successfully applied to *Klebsiella* and *Escherichia* species, reducing reliance on labor-intensive empirical host-range screening and facilitating personalized phage selection (Boeckaerts et al., 2024; Gaborieau et al., 2024).

Artificial intelligence (AI) has significantly advanced the computational discovery and characterization of therapeutic bacteriophages by enabling rapid analysis of large genomic and metagenomic datasets. AI-driven bioinformatic tools facilitate multiple stages of phage development, including identification of phage sequences, prediction of phage lifestyle, evolutionary analysis, and therapeutic phage selection. Deep learning-based platforms such as Seeker enable alignment-independent identification of phage sequences, including genetically diverse phages with limited similarity to known viral genomes (Auslander et al., 2020). Complementary tools such as PHACTS predict whether bacteriophages exhibit virulent or temperate lifestyles, supporting the selection of suitable candidates for therapeutic applications (McNair et al., 2012; Thung et al., 2021). Likewise, STEP^3^ employs ensemble machine-learning approaches to analyse phage genomic sequences and infer evolutionary characteristics, further improving phage characterization (Thung et al., 2021; Doud et al., 2025).

Evo 2 is one of the recent powerful advancements in AI-driven genomic analysis with significant implications for bacteriophage research. Evo 2 is trained on a large and diverse dataset that includes bacteriophage and host genomes. It is capable of capturing complex evolutionary patterns that are critical for understanding phage biology. One of its key strengths lies in predicting gene essentiality by modelling the effects of mutations, enabling the identification of genes that are crucial for phage survival and replication. This is particularly valuable in phage therapy, where distinguishing essential from non-essential genes can guide the selection of safe and effective therapeutic phages and support rational phage engineering (Brixi et al., 2026).

As AI continues to evolve, its integration into phage research is expected to further enhance predictive accuracy, therapeutic design, and translational efficiency. However, parallel efforts are required to standardize and integrate global phage repositories, ensuring interoperability and accessibility for clinical applications, including compassionate use and controlled trials. Such coordinated initiatives will be essential to fully realize the potential of AI-enabled phage therapy in addressing antimicrobial resistance (Doud et al., 2025).

## 8 Translational challenges and regulatory considerations in phage therapy

Despite encouraging preclinical and clinical outcomes, several scientific, manufacturing, and regulatory challenges continue to limit the routine clinical implementation of bacteriophage therapy. Therapeutic success depends on the identification of strictly lytic bacteriophages with broad activity against clinically relevant pathogens while avoiding temperate phages that may promote lysogenic conversion and horizontal transfer of virulence or antimicrobial resistance genes (Zalewska-Piątek, 2023; Gummalla et al., 2023).

Another major limitation is the emergence of phage-resistant bacterial populations through receptor modification or intracellular defense mechanisms, including CRISPR–Cas and restriction–modification systems (Nang et al., 2024; Fujiki et al., 2025; Urvoy et al., 2026). Current evidence indicates that phage cocktails targeting multiple bacterial receptors, together with personalized phage replacement strategies, represent the most practical approaches for delaying resistance development and maintaining therapeutic efficacy (Pires et al., 2020).

Host immune responses represent another translational consideration. Anti-phage antibodies, particularly IgM and IgG, may neutralize circulating phages and reduce therapeutic efficacy through accelerated clearance. Furthermore, phage proteins, nucleic acids, and residual bacterial contaminants can stimulate immune responses, emphasizing the need for highly purified preparations and careful immunological evaluation (Youssef et al., 2025; Washizaki et al., 2024). Large-scale manufacturing also remains challenging, as therapeutic phages must be produced according to stringent Good Manufacturing Practice (GMP) standards while ensuring quality, safety, potency, and consistency. However, dedicated manufacturing guidelines specific to phage products remain limited (Pirnay et al., 2024; Pires et al., 2020).

These scientific challenges are further compounded by regulatory uncertainty. Although bacteriophages are generally regulated as biological medicinal products, existing pharmaceutical frameworks do not adequately accommodate their biological complexity and personalized therapeutic application. Recent initiatives, including European Pharmacopoeia quality standards and regulatory pathways established under the United States Food and Drug Administration Investigational New Drug framework, represent important progress; however, globally harmonized regulations remain unavailable, restricting phage therapy largely to compassionate-use settings in several countries including the United States, Belgium, France, Poland, and Georgia (Fuerst-Wilmes et al., 2025; Moon et al., 2025; Niazi, 2025).

Future progress will require coordinated efforts among researchers, clinicians, industry, and regulatory authorities to establish robust manufacturing standards, generate high-quality clinical evidence, and develop regulatory pathways that accommodate the unique characteristics of bacteriophages as therapeutic agents. The recent development of standardized quality guidelines and regulatory initiatives reflects growing recognition of bacteriophages as viable therapeutic agents. Their integration with robust manufacturing practices and personalized therapeutic strategies will be pivotal in establishing bacteriophage therapy as a routine option for managing multidrug-resistant *Klebsiella pneumoniae* infections.

## 9 Conclusion

This review demonstrates that the clinical success of bacteriophage therapy against *Klebsiella pneumoniae* is determined not solely by phage-mediated antibacterial activity but by the coordinated interplay between host–phage immune interactions, therapeutic delivery strategies, phage-derived therapeutics, and translational implementation. Integrating these interconnected determinants provides a framework for optimizing phage persistence, bacterial clearance, and therapeutic efficacy while highlighting the importance of considering these factors collectively rather than independently. The growing shift toward precision phage therapy, supported by individualized phage selection based on pathogen susceptibility, host immune compatibility, and infection characteristics, together with engineered bacteriophages, phage-derived enzymes, and AI-assisted phage discovery, offers promising opportunities to enhance therapeutic performance against multidrug-resistant *Klebsiella pneumoniae*. Successful clinical translation will depend on integrating immune-guided therapeutic optimization, advanced delivery systems, standardized manufacturing, adaptive clinical trial designs, and harmonized regulatory frameworks through multidisciplinary collaboration across immunology, pharmacokinetics, genomics, microbiology, and regulatory science, thereby facilitating the establishment of precision bacteriophage therapy as a clinically reproducible and effective therapeutic strategy for multidrug-resistant *Klebsiella pneumoniae* infections.
